# Supplementary material for: Betholletia excelsa Fruit: Unveiling Toughening Mechanisms and Biomimetic Potential for Advanced Materials
Source: Biomimetics (Basel). 2023 Oct 26;8(7):509. doi: 10.3390/biomimetics8070509 (PMC10669686; doi:10.3390/biomimetics8070509)
Supplement: Supplementary file 1 [file biomimetics-08-00509-s001.zip › biomimetics-2592314-supplementary.pdf]

Supplementary information

Marilia. Sonego [1,2,*], Anneke Morgenthal [3], Claudia Fleck [3,†], Luis Antonio Pessan [1,4,†]

[1] Graduate Program in Materials Science and Engineering (PPGCEM), Federal University of São Carlos (UFSCar), São Carlos 13565-905, SP, Brazil; pessan@ufscar.br

[2] Institute of Mechanical Engineering, Federal University of Itajubá (UNIFEI), Itajubá 37500-903, MG, Brazil

[3] Materials Science and Engineering, Technische Universität Berlin, 10623 Berlin, Germany; claudia.fleck@tu-berlin.de (C.F.)

[4] Department of Materials Engineering, Federal University of São Carlos, São Carlos 13565-905, SP, Brazil

* Correspondence: mrl.sonego@unifei.edu.br

† These authors contributed equally to this work.

Materials and Methods

Table S1. Nomenclature, number of specimens, cut and loading direction and used in the mechanical tests. Each red line represents a cut

Test	Cut direction parallel to	Loading direction parallel to	Force type	Number of specimens	Specimen name
C-ring	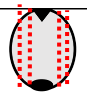 Longitudinal section	Longitudinal section	Compression	5	Longitudinal
			Tensile	5	Longitudinal
	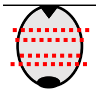 Latitudinal section	Latitudinal section	Compression	5	Latitudinal
			Tensile	5	Latitudinal
Compression of half mesocarp	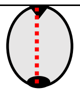 Longitudinal section	Latitudinal section	Compression	5	Latitudinal
	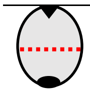 Latitudinal section	Longitudinal section		5	Longitudinal

C-ring

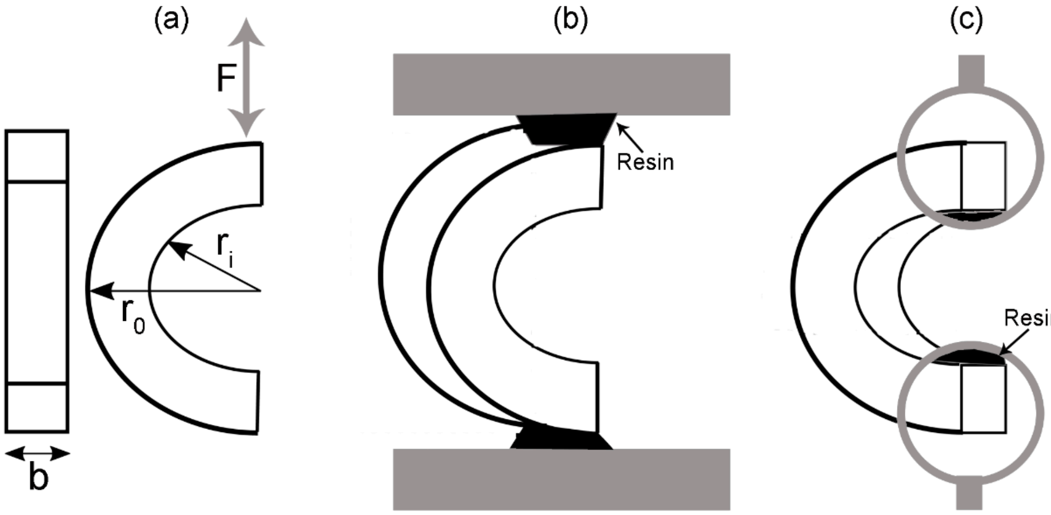

Figure S1: C-ring test: (a) specimen dimensions; (b) compression and (c) tension set up.

Elastic modulus (Equation (S1)) and fracture strength (Equation (S2) – tension and Equation (S3) - compression) were calculated from isotropic elastic beam theory for a semi-circular beam-shaped specimen with a rectangular cross-section .

$$E = \frac{3\pi\,(r_0 + r_i)^3}{4b\,(r_0 - r_i)^3} \cdot \frac{dP}{dx} \qquad \text{(S1)}$$

$$\sigma_{f,tension} = \frac{F}{b(r_0 - r_i)} \cdot \left(\frac{3(r_0 + r_i)}{r_0 - r_i} + 1\right) \qquad \text{(S2)}$$

$$\sigma_{f,compression} = \frac{F}{b(r_0 - r_i)} \cdot \left(\frac{3(r_0 + r_i)}{r_0 - r_i} - 1\right) \qquad \text{(S3)}$$

Where F is the force applied, x is the displacement of the cross-head, and all other variables are represented in Figure S1a.

Strictly speaking, Equations (S1)–(S3) are valid if Equation 4 is satisfied.

$$\frac{r_0 + r_i}{r_0 - r_i} \geq 20 \qquad \text{(S4)}$$

Applying the mesocarp dimension in Equation (S4) results in approximately 10; however, the equations were used anyway once a more accurate analysis would be complicated due to the natural variations of the shape of mesocarp, a biological structure. The same equations and arguments were used by Jennings and Macmillan [16] to test macadamia nutshell.

Compression of half mesocarp

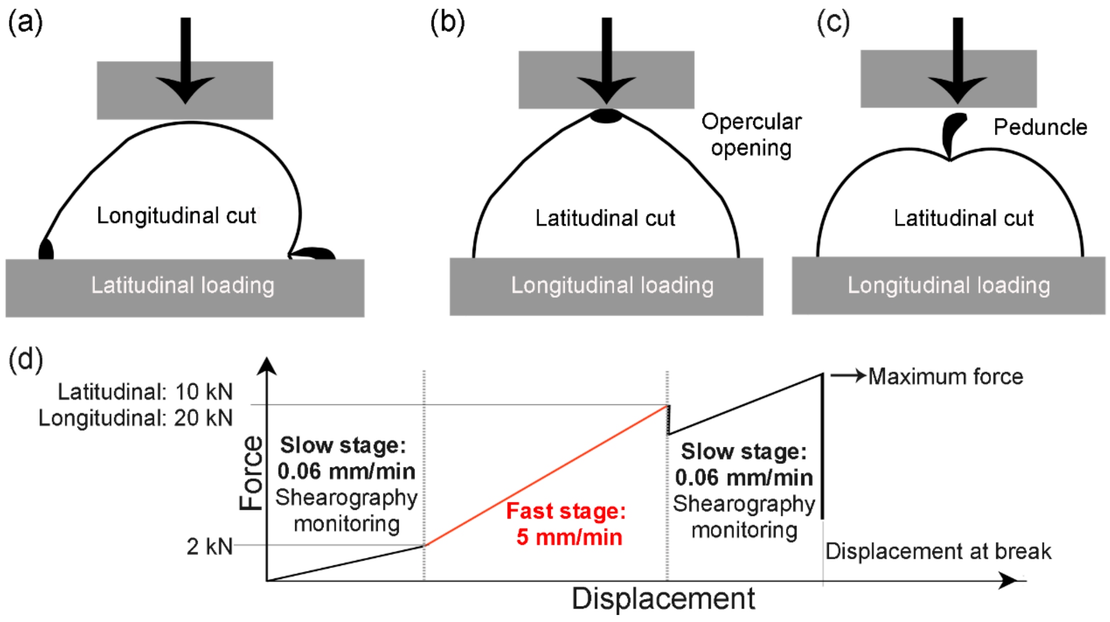

Figure S2: Half mesocarp specimens tested in compression test: (a) latitudinal and (b) longitudinal (with a peduncle or an opercular opening) specimens. Nomenclature was established based on the loading direction

Shearography

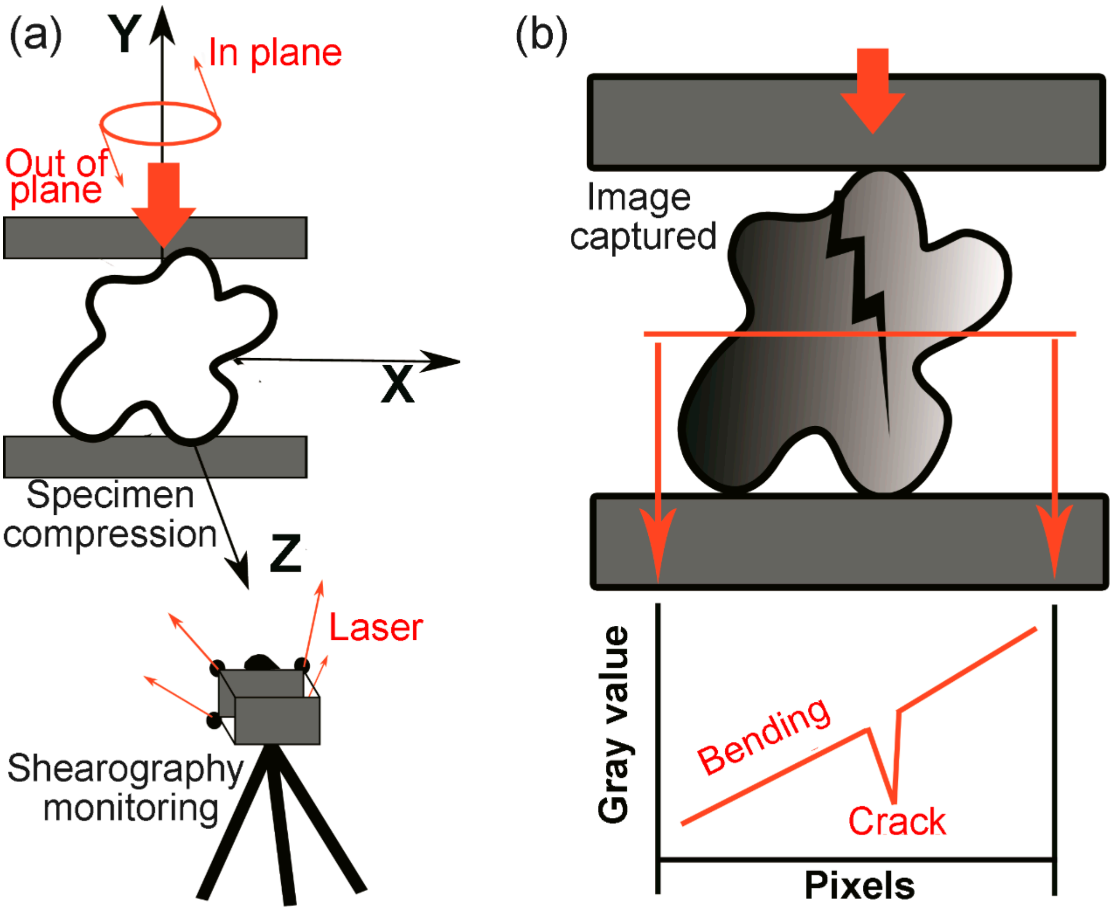

Figure S3: Shearography: Schematic drawing of (a) set-up to monitor the relative movement of rotation in and out of plane in the Y-axis –- (b) typical gray value distributions in a shearography image of a loaded mesocarp.

MicroCT

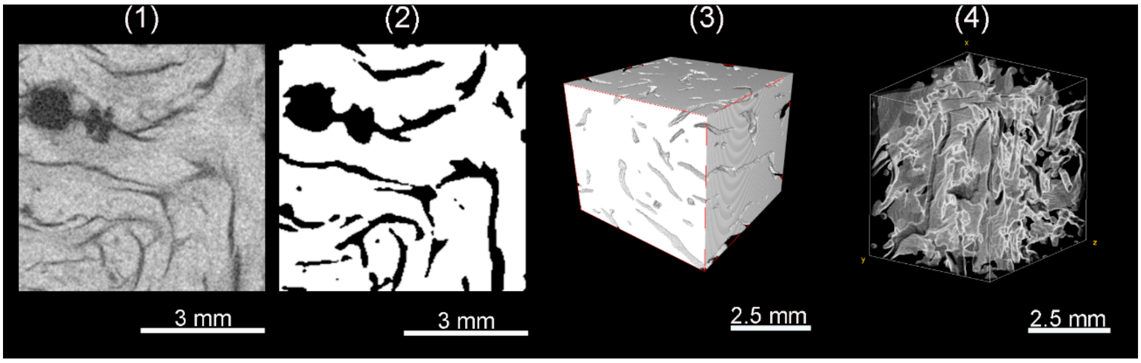

Figure S4: MicroCT images of mesocarp thickness: (1) 2D radiograph; (2) 2D segmented image with voids in black and cells in white (3) 3D model of the volume of interest in mesocarp structure and (4) 3D model of voids

FEM

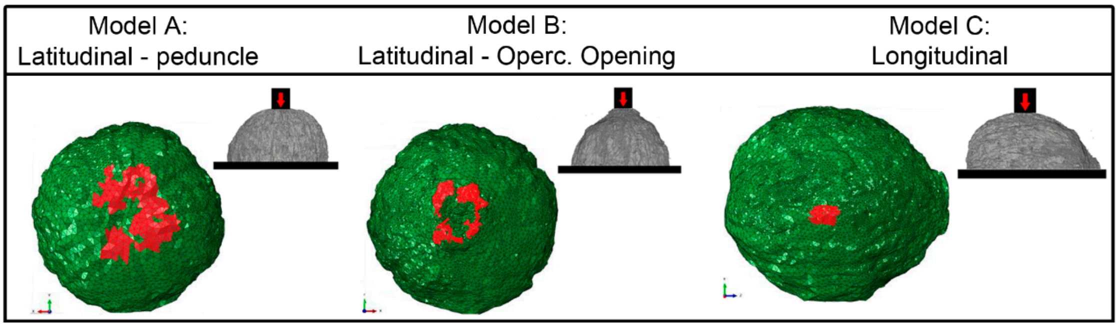

Figure S5: Loaded surfaces simulating contact area between compression tool and mesocarp at each FEM of compression test of half mesocarp

Results

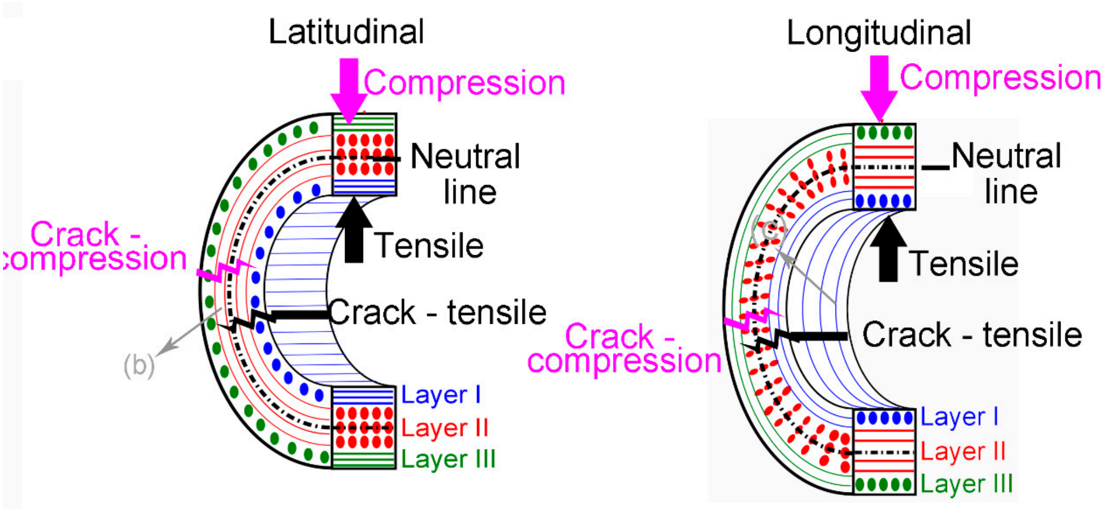

Figure S6: Schematic drawing of C-ring fractured specimens highlighting the load direction, fiber orientation and main crack path

Figure S6 shows a schematic drawing indicating the main crack path in the C-ring latitudinal and longitudinal specimens as well as its preferential fiber orientation. Under compression loading, the main crack propagates from layer III to layer I while it goes from layer I to layer III in the tensile test. Therefore, due to C-ring bending, the maximum tensile stresses in the tensile loading occur on the internal surface (layer I), and in compression loading, they are on the outer surface (layer III). Layer II, comprising the center part of the volume, experiences lower stresses in both loading configurations. In fact, the neutral line where the stress is zero, always passes through layer II.

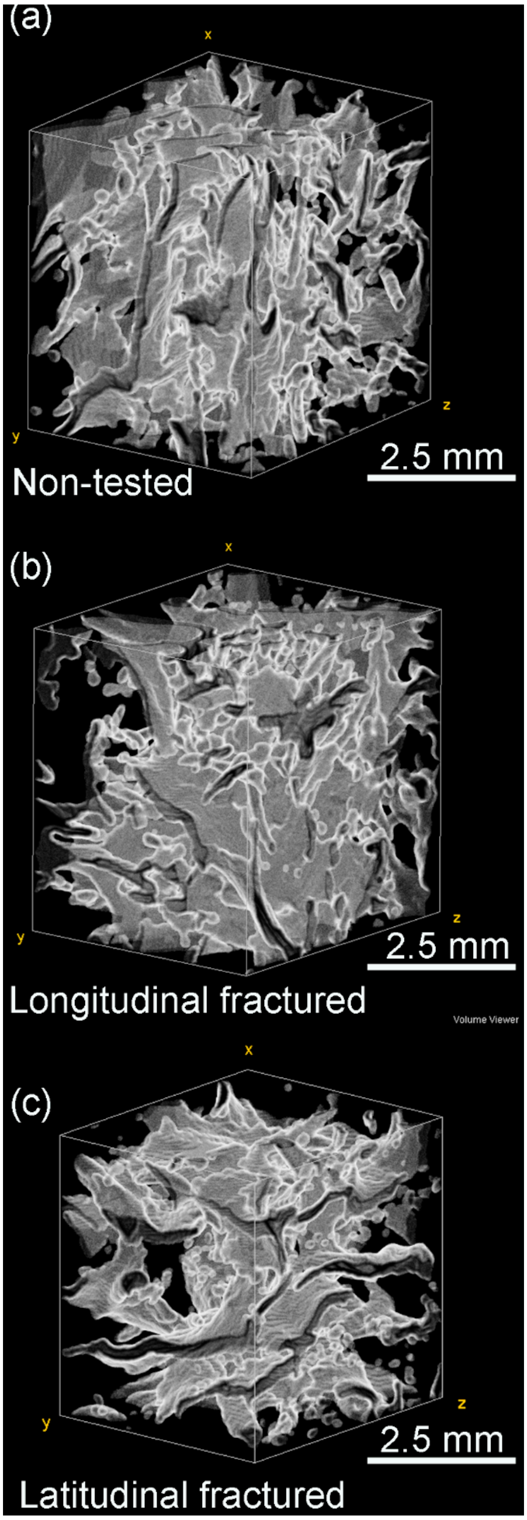

Figure S7: MicroCT 3D models of voids in a VOI from: (a) non-tested mesocarp, (b) longitudinal and (c) latitudinal specimens after compression loading
